# Quiescent Cancer Cells—A Potential Therapeutic Target to Overcome Tumor Resistance and Relapse

**DOI:** 10.3390/ijms24043762

**Published:** 2023-02-13

**Authors:** Emma Lindell, Lei Zhong, Xiaonan Zhang

**Affiliations:** Department of Immunology, Genetics and Pathology, Uppsala University, SE-751 85 Uppsala, Sweden

**Keywords:** quiescent, drug resistance, tumor recurrence, novel therapeutic strategies

## Abstract

Quiescent cancer cells (QCCs) are nonproliferating cells arrested in the G0 phase, characterized by ki67^low^ and p27^high^. QCCs avoid most chemotherapies, and some treatments could further lead to a higher proportion of QCCs in tumors. QCCs are also associated with cancer recurrence since they can re-enter a proliferative state when conditions are favorable. As QCCs lead to drug resistance and tumor recurrence, there is a great need to understand the characteristics of QCCs, decipher the mechanisms that regulate the proliferative–quiescent transition in cancer cells, and develop new strategies to eliminate QCCs residing in solid tumors. In this review, we discussed the mechanisms of QCC-induced drug resistance and tumor recurrence. We also discussed therapeutic strategies to overcome resistance and relapse by targeting QCCs, including (i) identifying reactive quiescent cancer cells and removing them via cell-cycle-dependent anticancer reagents; (ii) modulating the quiescence-to-proliferation switch; and (iii) eliminating QCCs by targeting their unique features. It is believed that the simultaneous co-targeting of proliferating and quiescent cancer cells may ultimately lead to the development of more effective therapeutic strategies for the treatment of solid tumors.

## 1. Introduction

Solid tumors represent almost 90% of adult cancers [1]; unfortunately, despite the success of standard treatments, most patients eventually recur, with ultimately chemo-resistant disease. Tumors consist of large numbers of heterogeneous cancer cells that differ in their genetic makeup, proliferation rate, and response to various therapies. The population of quiescent cancer cells (QCCs, ‘sleeping’, slowly proliferating, nonproliferating, and cell-cycle-inactive) residing in areas far from blood vessels is considered an important reason for cancer drug treatment failure in solid tumors [2]. QCCs have been identified in various cancer types, such as glioblastoma [3], lung cancer [4], breast cancer [5], colorectal cancer [6], and skin cancer [7]. In contrast to senescent cells that are irreversible in cell cycle arrest [8], QCCs are temporarily and reversibly arrested in the G0 phase [9]. Compared with cancer stem cells (CSCs), QCCs share several common features, such as drug resistance, rarity, and an ability to cause cancer recurrence. However, QCCs are fundamentally different from cancer stem cells. CSCs have the characteristics of self-renewal, pluripotency, and high plasticity [10], and exist in any stage of the cell cycle [11,12]. QCCs are only cells present in the nonproliferating G0 phase [13]. In this review, we discuss the reported markers and their function in quiescence induction, mechanisms of QCC-induced drug resistance, and tumor recurrence. We also review recent therapeutic strategies targeting QCCs to overcome drug resistance and relapse in cancer therapy.

## 2. Quiescent Cancer Cells and Their Regulation

The induction of a quiescent state (G0 phase) is dependent on diverse factors, such as inducing signals in the microenvironment, a loss of cell adhesion and cell contact, nutrition starvation, and hypoxia [13,14]. The inducing signals include the expression of the cell cycle suppression proteins—for example, p27 and p21—and the decreased expression of cell cycle activation proteins such as CDKs and the hypo-phosphorylation of Rb proteins. For instance, the upregulated expression of p27, resulting in the suppression of G1 CDK 4/6, is observed during serum starvation [15], playing a significant role in the conversion between a proliferating and quiescent state. To identify QCCs, high expression of p27 and low expression of the proliferation marker ki67 are usually used together as a criterion [16,17]. In addition to cell cycle regulators p27^high^ and ki67^low^, the quiescent state could also be controlled by other factors, such as the dormancy factors ERK1/2^high^/p38^low^ [18], NR2F1 [19], and dual-specificity tyrosine phosphorylation-regulated kinase 1A (DYRK1A) and 1B (DYRK1B). DYRK1A is involved in arresting cells in the G0 phase by activating the DP, Rb, E2F, and MuvB (DREAM complex), which could further suppress the expression of cell cycle genes [20]. In some studies, QCCs were also recognized by 2N DNA content with low RNA content [21], the presence of biochemical markers including the p130/Rb2 binding complex [22], and a lower amount of mitochondrial content [23]. However, mitochondrial oxidative phosphorylation (OXPHOS) activity may be elevated and significantly associated with the survival of QCCs [16]. Table 1 provides the list of markers used to define quiescent cancer cells.

## 3. Quiescent Cancer Cells Are Resistant to Chemotherapy and Immunotherapy

**Figure 1:**
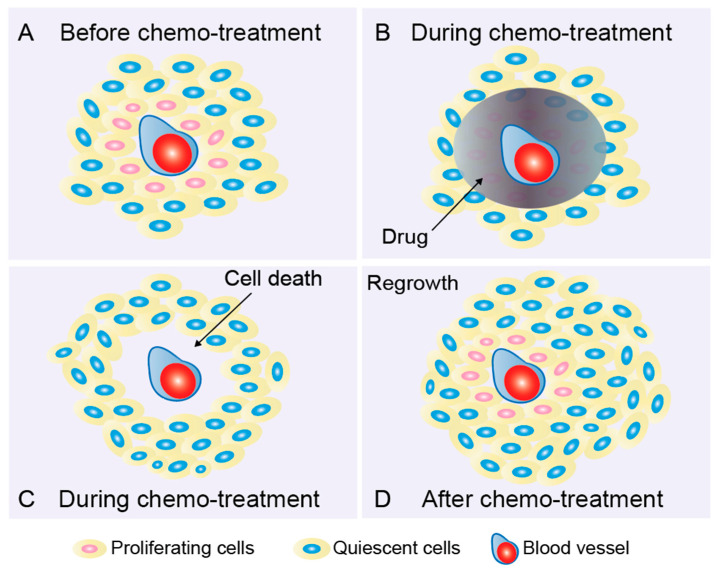
Quiescent cancer cells lead to drug resistance and tumor recurrence. (**A**) Quiescent cancer cells (QCC, “dormant”, slow-proliferating, nonproliferating, and cell-cycle-inactive) located in areas far from blood vessels are considered to be an important cause of treatment failure in solid tumors. QCCs proliferate slowly and are insensitive to cell-cycle-active anticancer drugs. During the cycle of treatment, proliferating cells (red) sufficiently exposed to the drug (**B**) are removed; however, QCCs (blue) that are not properly exposed to the drug and inherently treatment-insensitive are spared (**C**). These QCCs are able to repopulate between treatment cycles when microenvironments are favorable (**D**). This process is defined as tumor recurrence or relapse. It has been observed that, under favorable conditions, tumors enriched in chemoresistant QCCs recur significantly earlier than tumors bearing fewer QCCs [36].

It is a clinical fact that despite the success of standard treatments such as surgery and chemotherapy, most patients eventually recur, with ultimately chemo-resistant disease, and the causes of tumor resistance and relapse may be complex. Pharmacokinetic disturbances caused by poor vascular tissue are one possibility [26], and QCCs located in remote vascular regions are another indispensable contributor [27]. Nowadays, most anticancer agents used in the clinic are designed to target proliferating cells (S/G2/M), making the phase of the cell cycle an important determinant of whether a cancer cell will respond to a given drug. However, more than 80% of internal cancer cells within a tumor have been reported to be in a quiescent state, ultimately leading to the ineffective elimination of solid tumors [28] (Figure 1). Notably, studies have also shown that anticancer therapy can increase the proportion of QCCs, thereby further enhancing chemoresistance after treatment cycles. Yano et al. reported that the proportion of quiescent cancer cells in subcutaneous tumors was increased compared with controls at 7 days after three cycles of cisplatin, paclitaxel, or doxorubicin, and the time to recurrence was positively correlated with the quiescent cancer cell frequency [29]. A similar phenomenon was also noted when cancer cells were exposed to 5-fluorouracil [30], gemcitabine [25], or radiation [31]. In another study, Kabraji et al. compared the percentage of QCCs in primary triple-negative breast tumor samples before and after multidrug, multicycle neoadjuvant chemotherapy. Consistent with the findings of Yano et al., the proportion of QCCs was increased in post-treatment mastectomy specimens compared with pre-treatment biopsies, but Kabraji et al. also pointed out that this finding should be further validated in larger cohorts [32].

The existence of QCCs is also a huge challenge in tumor immunotherapy [33]. Only recently, Agudo’s group at Harvard’s Ludwig Center found that in primary triple-negative breast tumor cells, cells that resist T cell attack are cell-cycle-inactive (QCCs). QCCs could form immune-infiltrated reduced clusters and express higher levels of chemoresistance and stemness genes. Single-cell RNA sequencing with precise spatial resolution to profile infiltrating cells inside and outside the QCC niche further revealed upregulated hypoxia-induced signals and more exhausted T cells within QCC clusters, suggesting that QCCs constitute a reservoir of immunotherapy resistance by coordinating the blockage of T cell function in a hypoxic immunosuppressive microenvironment [34]. Another study on myeloid-derived suppressor cells, the key cellular suppressors of antitumor immune responses, suggested that tumor cells with a low expression level of proliferation marker ki67 are susceptible to immuno-editing and escape during cell division [35]. Although there are few reports on the relationship between QCCs and resistance to immunotherapy, QCCs undoubtedly play an important role, which deserves further study.

## 4. Quiescent Cancer Cells Lead to Tumor Recurrence

After initial treatment with surgical, chemical, or radiotherapies, patients may be considered clinically cancer-free, but, unfortunately, the possibility of recurrence at the primary or distant metastatic site emerges months or years later. Cancer cells that metastasize to distant organs are initially quiescent, viable but inactivated by reversible G0-G1 phase arrest. These QCCs are more resistant to antiproliferative drugs and gradually adapt to the new microenvironment at the primary or the metastatic site. The QCCs can then re-enter the cell cycle when conditions permit. This process is defined as tumor recurrence or relapse [37] (Figure 1). Tumor recurrence is a complex and poorly understood phenomenon that unfortunately limits our ability to fully cure cancer. The definition of tumor recurrence suggests that QCCs play an important role. In fact, it was noticed that tumors enriched in chemoresistant QCCs relapsed significantly earlier than tumors harboring fewer QCCs under favorable conditions [36]. Another study revealed that QCCs are able to induce nascent vessels in the deeper areas of the tumors after chemotherapy, which allows tumor regrowth after cessation of treatment, providing a strong explanation for the observation that tumors enriched in chemoresistant QCCs relapse significantly earlier than tumors harboring fewer QCCs [29]. In addition, Kabraji et al. reported that QCCs are found in triple-negative breast tumor deposits within the brain, axillary lymph nodes, and skin [32]. Disseminated tumor cells (DTCs) in the bone marrow can remain viable but are growth-arrested until re-entering the cell cycle, producing late metastases after years of apparently disease-free survival [38]. The presence of DTC in the bone marrow has also been shown to be associated with a higher risk of clinically detectable breast cancer metastasis [39,40]. A similar finding has also been reported in cancer patients with colon liver metastases [41] and MYC-amplified high-risk medulloblastoma [42]. In short, findings discussed here suggest that QCCs may exist earlier than relapse and play a key role in tumor recurrence. However, it should be pointed out that the quiescent state has recently been described as a heterogenous state, both in normal cells and in QCCs, depending on the induction signals in the microenvironment. The study by Coller et al. presented that, depending on the induction signal, loss of adhesion, mitogen starvation, or contact inhibition, the quiescent cells display various gene expression profiles. This initiates the expression of distinct cell cycle exit signals and causes the quiescent cells to appear in different quiescent states [43]. Recently, one study compared the expression profiles of QCCs isolated from non-small-cell lung cancer (NSCLC) and colorectal cancer (CRC) tumor xenografts. They identified a similar main quiescence-associated transcription program between both tumors that underlined an upregulation in stemness, pro-metastatic, chemoresistance, and EMT-like pathways, e.g., KLF4 and ZEB2 [44]. Moreover, in addition to ki67 and p27, researchers also consider other markers to focus on specific proportions of QCCs, e.g., Yes and YAP [41] or SOX9 [42], revealing the diversity of QCCs across different conditions (e.g., primary site, distant metastatic site, cancer type, or specific genomic signature).

## 5. Targeting QCCs to Overcome Resistance and Relapse in Cancer Therapy

The ability of QCCs to evade chemotherapy, exist undetected for years before cells resume division, and ultimately lead to treatment failure in solid tumors suggests that QCCs pose a very high potential risk in tumor recurrence and may mislead the post-treatment assessments. Considering all the obstacles posed by QCCs to the treatment of solid tumors, there is a great need to understand the characteristics of QCCs, decipher the mechanisms that regulate the proliferative–quiescent transition in cancer cells, and develop new strategies to eliminate QCCs residing in solid tumors. This remains a long-standing challenge in the future.

Karla Santos de Frutos and Nabil Djouder have addressed an important discussion in the treatment of QCCs and described three different strategies to target quiescent cells: (1) the “awakening” of dormant quiescent cells; (2) keeping cells in a quiescent state; and (3) removing cells while quiescent [45]. Here, based on their extensive work and findings from other studies, we present our strategies and discuss them below.

### 5.1. Strategy 1: Reactivate Quiescent Cancer Cells and Remove Them via Cell-Cycle-Dependent Anticancer Reagents

Most drugs currently used in the clinic are designed to target cancer cells in their proliferative phases (S/G2/M). Therefore, cancer cells in the quiescent state are insensitive to these cell cycle-dependent drugs. Due to this reason, strategies to reactivate quiescent cancer cells to rapidly re-enter the cell cycle hold promise in improving the elimination of QCCs via antiproliferative drugs. In fact, this strategy is supported by different research findings. For example, studies on quiescent cancer stem cells have shown that cell proliferation can be induced when pretreated with IFNα and STAT1 and these cells could be further targeted by 5-Fluorouracil (5-FU) [46]. The induction of the transition from quiescent to proliferative status may also require the activation of the integrin β1 (ITGB1) receptor or signaling through the activation of focal adhesion kinase (FAK), Src, ERK1/2, and MLCK [47]. Another interesting finding was reported by Yano et al. They demonstrated that S. typhimurium A1-R can convert cancer cells from the quiescent phase to the cell-cycle-active S/G2/M phase, thereby re-sensitizing them to cisplatinum or paclitaxel [48]. S. typhimurium is a facultative anaerobe. Compared to obligate anaerobes, facultative anaerobes have important advantages. For example, facultative anaerobes can grow in the oxic viable regions as well as the necrotic regions of tumors [49]. Based on the dominance of facultative anaerobes, Yano et al. developed a mutant strain, S. typhimurium A1-R, which is auxotrophic for Leu-Arg, thus unable to create a persistent infection in normal tissues. At the same time, S. typhimurium A1-R exhibited high tumor colonization efficacy and antitumor efficacy [50,51,52]. Further results indicated that before S. typhimurium A1-R treatment, approximately 95% of the cancer cells were quiescent; interestingly, after S. typhimurium A1-R treatment, the proportion of QCCs was significantly reduced to approximately 30%, and proliferating cells accounted for the remaining 70%. The combination of S. typhimurium A1-R with cisplatinum or paclitaxel resulted in greater growth inhibition compared with cisplatinum or paclitaxel monotherapy [48]. Studies also indicated that miRNAs could awaken QCCs. miRNA-27b-3p and miRNA-455-3p are two p53-responsive miRNAs that were expressed at higher levels in quiescent cells compared to proliferating cells. The further inhibition of miRNA-27b-3p or miRNA-455-3p resulted in a decrease in the proportion of quiescent cells in tumors, suggesting that manipulating the expression levels of miRNA-27b-3p or miRNA-455-3p may reactivate QCCs into a proliferative state, which may be further targeted by cell-cycle-dependent anticancer agents [53]. In another study, Almog et al. applied miRNA profiling to characterize the unique expression pattern of QCCs in osteosarcoma and glioblastoma. They found that miR-190 was one of the most upregulated miRNAs in the QCC population. They further generated a cell line from U87-miR-190 tumors that “escaped” quiescence to become rapidly growing and aggressive tumors. This generated cell line exhibited significantly lower levels of miR-190 than the parental U87-miR-190 cell line, suggesting that the reduction of miR-190 can drive QCCs into a cell-cycle-active state [54]. In addition, miR-138 or miR-346 can also promote QCCs from a quiescent state to a proliferative state in metastatic lesions and mediate the metastatic reactivation of breast cancer in mice [55], suggesting that modulating the expression levels of miRNAs could stimulate a transition from quiescence to proliferation. Basal cell carcinoma, the most common cancer in humans, results from the constitutive activation of the Hedgehog pathway [56]. Vismodegib, a smoothened inhibitor (Smoi), causes basal cell carcinoma shrinkage in most patients; however, a small fraction of tumor cells persists and causes tumor recurrence when treatment is stopped [57,58]. Recently, researchers discovered that this persistent population of cancer cells is slow-cycling and has a high expression level of Lgr5 and more active Wnt signaling. Inhibition of Wnt signaling with a porcupine inhibitor LGK-974, together with vismodegib, led to tumor eradication [7], constituting a clinically relevant strategy to overcome tumor relapse by reactive quiescent cancer cells through the inhibition of Wnt signaling.

It is worth mentioning that, in some cases, unfortunately, chemotherapy followed by the induction of cell proliferation failed to improve or even worsened the treatment outcome [59]. Since this strategy could awaken cells that should not be active, it increases the risk of tumors developing elsewhere. At the same time, there is no guarantee that all cancer cells moving from a quiescent state to a proliferative state will be completely eliminated. For example, TGF-β2 was identified as a key inducer of quiescence in head and neck cancer cell lines. In a mouse model, inhibition of TGF-β receptors with LY-364947 led to QCC reactivation and an increased metastatic burden in the liver, spleen, and bone marrow [59]. Overall, strategies to awaken QCCs are promising but also challenging.

### 5.2. Strategy 2: Modulate the Quiescence-to-Proliferation Switch

Another strategy to overcome tumor resistance and relapse in cancer therapy is modulating the quiescence-to-proliferation switch to make QCCs continuously quiescent.

To achieve this, one possible approach is to understand and mimic the mechanisms of quiescence induction and thus to avoid rapid proliferation and tumor regrowth. In 2011, Dey-Guha et al. discovered that in MCF7 breast, MDA-MB-231 breast, and HCT116 colon cancer cell lines, rapidly proliferating cells can divide asymmetrically to generate a proportion of “G0-like” quiescent cancer cells. Further studies showed that MCF7 and MDA-MB-231 breast cancer cells could form a quiescent, AKT1^low^ cell population (QCCs) to respond to the reduction of integrin-β1 (ITGB1) signaling from the microenvironment. These AKT1^low^ QCCs cells also exhibited low levels of ROS, MKI67, MCM2, and H3K9me2, and a high level of HES1, but did not alter the expression of ESR1 or MYC [60,61]. Furthermore, patients with abnormal tumors harboring the AKT1-E17K mutation and unable to generate AKT1^low^ QCCs showed significantly longer survival after initial treatment compared with other patients [32]. A similar finding of modulated AKT signaling was also reported for the treatment of ovarian cancer cells [47], indicating that modulating AKT signaling could in fact regulate the proportion of QCCs in tumors. However, it should be pointed out that drugs targeting AKT signaling directly or indirectly can promote the formation of AKT1^low^ QCCs, but cannot eliminate them [62,63], and the issue of how to maintain QCCs for a long time without drug resistance and tumor relapse is still a challenge. With the exception of targeting AKT signaling, studies also indicated that modulating the p38/ERK pathways could lead to the permanent growth arrest of quiescent cells (senescent), thus preventing tumor recurrence and metastasis [64]. In addition, the inhibition of MET kinase activity could prevent the downstream phosphorylation of ERK1/2 and AKT, and further lead to cell cycle arrest. However, these QCCs were shown to be able to resume growth after the release of the MET blockade, suggesting that continued treatment is required to maintain the quiescent status of QCCs [65,66].

The other possible approach is to understand the mechanism of the quiescence-to-proliferation transition and to further suppress stimuli or signals that have been identified to be involved in the cell cycle activation process.

(a) Target Src family kinase

Touny et al. investigated the role of Src family kinase (SFK) in regulating the quiescence-to-proliferation switch and found that pharmacological inhibition of SFK signaling or *Src* knockdown prevents the proliferative outbreak of quiescent breast cancer cells and metastatic lesion formation [67]. Since QCC proliferation also requires the activation of ERK1/2 [47,68], inhibiting ERK1/2 in combination with the SFK inhibitor AZD0530 resulted in the apoptotic death of quiescence cells, which does not occur when using either agent alone [67].

(b) Target non-canonical discoidin domain receptor 1 signaling

In addition, the activation of non-canonical discoidin domain receptor 1 (DDR) signaling is known to stimulate cancer cell proliferation at metastatic sites [69,70]. The whole process is complicated. In short, tetraspanin 4L six family member 1 (TM4SF1) requires the coupling of DDR1 and syntenin 2 to activate protein kinase Cα (PKCα). Activated PKCα then phosphorylates Janus kinase 2 (JAK2), which drives non-canonical DDR1 signaling through the phosphorylation of signal transducers and activators of transcription 3 (STAT3), further upregulating the transcription of cell cycle regulators, such as c-Myc and cyclin D, and promoting cancer cell proliferation. Therefore, inhibition of any step in this activation process may prolong QCCs’ status and delay tumor recurrence.

(c) Target Wnt signaling

Wnt signaling controls a variety of biological processes and functions, and its upregulation is another well-known inducer of proliferation. Studies have revealed that activated Wnt signals can promote G1-to-S progression through the regulation of cyclin D1, cyclin E1, and c-myc [71,72]. Therefore, the inhibition of Wnt signaling could be another means to maintain QCCs’ status and thus prevent tumor relapse. In fact, the expression of the Wnt inhibitor Dickkopf-related protein 1 (DKK1) has been reported to induce cancer cells into quiescence [5].

(d) Target cyclin-dependent kinases

Modulating the expression levels of cyclin-dependent kinases also manipulates the cancer cell state. Cyclin-dependent kinases (CDKs) are a group of serine/threonine kinases that form complexes with cyclins to stabilize, activate, and phosphorylate their target genes, control cell cycle checkpoints and transcription, and regulate cellular proliferation [73,74,75]. Given their important roles in cell growth, the inhibition of cyclins and CDKs may be another possible approach to maintain QCC status and delay tumor recurrence. For instance, CDK4 and CDK6 are frequently activated in human cancer, either by overexpression of their activating subunits, D-type cyclins, or inactivation of CDK4/6 inhibitors of the INK4 protein family, and they play an essential role in cell cycle entry and G1 progression [76,77,78]. Inhibition of the cell cycle kinases CDK4 and CDK6 is now part of the standard treatment in advanced breast cancer; recently, a study has revealed that the sequential administration of CDK4/6 inhibitors after taxanes cooperates to prevent cell cycle re-entry in pancreatic ductal adenocarcinoma (PDAC) [79], indicating that cell-cycle-dependent kinases could be a potential target in preventing the quiescence-to-proliferation switch.

(e) Target proto-oncogene MYC family

The proto-oncogene MYC (MYC, MYCL, and MYCN) is a well-defined regulator of key cellular processes including cell proliferation and the cell cycle, and can promote re-entry into a mitotic state at high expression levels [80,81]. Studies revealed that a CPF mixture (Coptis chinensis, Pinellia ternate, and Fructus trichosanthis) could induce the transcriptional suppression of c-MYC genes and thus impede the quiescence-to-proliferation switch in lung cancer cells [82]. Guttiferone K is a bioactive polycyclic polyisoprene phloroglucinol that prevents cell cycle re-entry by promoting c-MYC protein degradation. The researchers noticed that under vector-treated conditions, the cells were able to restore regrowth by upregulating the expression levels of c-MYC. However, when treated with Guttiferone K, the c-MYC protein was degraded by the stabilization of FBXW7, and cell cycle re-entry was hindered in prostate cancer cells, suggesting that manipulating the protein levels of MYC could further regulate the quiescent-to-proliferative switch [83,84].

(f) Target chronic inflammation

It is noteworthy that chronic inflammation can also promote the reactivation of QCCs, and a strong correlation between inflammation and recurrence of cancer has been demonstrated in several studies [85,86]. After treatment, the upregulation of serum inflammatory markers C-reactive protein (CRP) and interleukin-6 (IL-6) predicts a high risk of cancer recurrence and patient death [87,88]. Likewise, local inflammation in the lung triggers the escape of cancer cells from a quiescent state, leading to the development of macroscopic metastases. During this process, upregulation of the expression of Zeb1, a strong inducer of epithelial to mesenchymal transition (EMT), is required [89,90]. Although, more conclusive evidence is still needed to explain the connection between chronic inflammation and the quiescence-to-proliferation switch, avoiding or alleviating local inflammation should be considered as a strategy to inhibit QCC activation and thus prevent tumor recurrence.

Many valuable discoveries have been reported to prevent the transition from quiescence to proliferation, some of which have shown great efficacy in preclinical models [67,79]. Investigations into chronic inflammation may contribute to the development of new therapeutic strategies to overcome QCCs’ cell cycle re-entry and thereby prevent tumor recurrence. However, despite encouraging results, there are significant limitations and challenges. If we do not remove the QCCs, we need to allow them to rest for a long time to prevent tumor regrowth. This means that we need to overcome the high adaptability of cancer cells to different situations, the toxic side effects of a given treatment, and eventually the inevitable drug resistance, which does not seem to be clinically feasible at this stage.

### 5.3. Strategy 3: Eliminate QCCs by Targeting Their Unique Features

The optimal outcome of cancer treatment is the eradication of all cancer cells, both proliferating and quiescent cancer cells. Since QCCs have different characteristics compared to proliferating cells, efforts to discover these differences and develop strategies to eradicate QCCs are encouraged. We collected several characteristics; however, by no means can we cover them all, as they have been studied separately in different studies.

(a) Quiescent cancer cells exhibit altered mitochondrial activity

Recently, several studies have demonstrated that the inhibition of mitochondrial OXPHOS is a promising strategy to combat quiescent cancer cells in hypoxic and nutrition-compromised environments. La et al. developed a melanoma cell model in which the endogenous cell-cycle-inactive marker p27 provides enhanced GFP signaling and the endogenous cell-cycle-active marker ki67 provides enhanced mCherry signaling. Using this cell model, the authors identified a group of cancer cells expressing low levels of ki67 and high levels of p27 (p27^high^ and ki67^low^), which are considered to be in a quiescent state. Compared to other cells, these QCCs exhibited a high expression level of c-Myc and stimulated activity of mitochondrial OXPHOS by transactivating genes encoding OXPHOS enzymes, including subunits of isocitric dehydrogenase 3 (IDH3). Further inhibition of mitochondrial OXPHOS by the small-molecule inhibitor of mitochondrial complex I, IACS-010759 [91], could lead to reduced cell viability in quiescent cells, whereas they do not significantly affect the viability of cell-cycle-active cells [16], suggesting that the targeting of mitochondrial OXPHOS can overcome drug resistance in QCCs. Similar findings have also been reported in other studies. For example, using a glucose-deprived multicellular tumor spheroid (MCTS) model with a population of QCCs in the core, Senkowski et al. screened 1600 compounds with a documented clinical history and identified five molecules showing pronounced MCTS-selective activity: nitazoxanide, niclosamide, closantel, pyrvinium pamoate, and salinomycin. Further experiments revealed that all five identified compounds inhibited mitochondrial respiration, suggesting that MCTS containing populations of QCCs depend on oxidative phosphorylation rather than only glycolysis [92]. In another study, authors established three distinct models (monolayer, proliferative-MCTS, and quiescent-MCTS) using colon carcinoma HCT116 cancer cells and profiled gene expression on a panel of compounds targeting various processes (mitochondrial inhibitors, autophagy inhibitor, kinase inhibitors, mTOR inhibitors, MEK inhibitors, etc.). The authors further discovered that upon exposure to OXPHOS inhibitors, the mevalonate pathway was significantly upregulated. A combination of cholesterol synthesis inhibitor zaragozic acid with mitochondrial inhibitor nitazoxanide resulted in a strong reduction in colony formation. However, the combination of nitazoxanide with irinotecan, dual PI3K/mTOR inhibitor BEZ235, or an inhibitor of autophagy Lys05 did not lead to increased toxicity towards quiescent-MCTS, suggesting that inhibition of the mevalonate pathway is a promising strategy to potentiate the effects of OXPHOS inhibitors against QCCs [93]. Interestingly, it has also been shown that impairing mitochondrial fatty acid β-oxidation (FAO) can induce apoptosis in quiescence-induced cells and hamper their return to proliferation, suggesting that targeting mitochondrial metabolism in QCCs could reveal fundamental principles in cell plasticity and new potential therapeutic options [94]. We also reported that VLX600, a mitochondrial inhibitor, is able to eliminate not only proliferative but also quiescent cancer cells, due to the induction of a bioenergetic catastrophe after mitochondrial inhibition [95]. Mitochondria are the main source of ATP production and also play important roles in building macromolecules, regulating signaling processes, maintaining ROS homeostasis, regulating intrinsic cell apoptosis, and cancer metastasis [96,97]. Therefore, it is reasonable to suggest that mitochondria should be indispensable in QCCs, and targeting mitochondria, such as OXPHOS, may be a promising strategy to eliminate QCCs [16,91,92,93,98]. However, many issues are not yet understood. For example, which complex on the mitochondrial electron transport chain is more important in QCCs? Which is the key metabolic pathway—OXPHOS, glycolysis, or lipid metabolism? What is the role of mtDNA in QCCs? etc. The answers to these questions are more likely to provide new therapeutic strategies for eliminating QCCs in solid tumors.

(b) Quiescent cancer cells cannot tolerate aggravated autophagy

In solid tumors, quiescent cancer cells are often located in areas away from blood vessels that are starved of nutrients and oxygen. Our previous studies showed that VLX600 exhibited extreme toxicity to quiescent cancer cells due to its mitochondrial inhibition and induction of autophagy [95]. In another study, quiescent cancer cells treated with a ULK1 inhibitor, a key kinase that activates autophagy, in combination with standard chemotherapy treatment (CPT-11), underwent apoptosis and were unable to re-grow after treatment [99]. Another example is saikosaponin A, a Bupleurum-derived compound that was able to further exacerbate autophagy by inactivating Akt-mTOR signaling and effectively eliminate multidrug-resistant quiescent prostate cancer cells. In addition, administration of saikosaponin A during the docetaxel treatment interval resulted in robust cell death in vitro and in vivo, suggesting that saikosaponin A may enhance therapeutic efficacy and prevent cancer recurrence by targeting QCCs [100].

(c) Quiescent cancer cells show high levels of DYRK1B

The dual-specificity tyrosine-regulated kinase (DYRK) family, including DYRK1A, DYRK1B, DYRK2, DYRK3, and DYRK4, belongs to the CMGC group, which includes cyclin-dependent kinases (CDKs), mitogen-activated protein kinase (MAPK), glycogen synthase kinase (GSK), and CDK-like kinases (CLKs) [101]. Family member DYRK1B has demonstrated a strong increase when tumor cells exit the cell cycle upon mitogen deprivation or the pharmacological inhibition of proliferative pathways in different cancer cell types (breast, colon carcinoma, melanoma, pancreatic, and ovarian cancer cells) [102,103,104]. On the contrary, decreasing the level of DYRK1B by RNA interference enables C2C12 myoblasts to re-enter the cell cycle [105], suggesting that DYRK1B plays an important role in maintaining cancer cells in a quiescent state. The underlying mechanism could be complex, but some evidence has suggested that DYRK1B is able to control the S-phase checkpoint by stabilizing the cyclin-dependent kinase inhibitor p27Kip1 and inducing the degradation of cyclin D [24,106]. DYRK1B also stabilizes the DREAM complex (DP, RB, E2F, and MuvB), an essential coordinator in maintaining the cell’s quiescent G0 state, by phosphorylating LIN52 on Ser28 [107]. In addition, DYRK1B has a pro-survival function by upregulating the expression of antioxidant genes and reducing the level of intracellular reactive oxygen species [108,109]. Substantial evidence indicates that the depletion or inhibition of DYRK1B drives cell cycle re-entry and enhances the apoptosis of those quiescent cancer cells with high expression of DYRK1B [110,111,112]. Furthermore, DYRK1B inhibitors were shown to sensitize cells to the cytotoxic effects of anticancer drugs that target proliferating cells [113,114]. In conclusion, targeting upregulated DYRK1B levels in QCCs could disrupt the quiescent status and further eliminate them via anticancer reagents that target proliferating cells.

(d) Quiescent cancer cells show the upregulation of the c-YES/YAP signaling axis

c-YES, a cytoplasmic non-receptor protein belonging to the SRC kinase family (SFK), has been shown to have oncogenic properties and act as a biomarker in different tumor types [115]. c-YES is overexpressed in cancer cells and associated with poor prognosis [41,116]. Amplification of c-YES also occurs in some EGFR and ALK inhibitor-treated patients who become resistant to targeted therapy [117,118]. Recent studies showed that, in the HT29 colon cancer cell line, 5FU-resistant clonal cell populations could enter a reversible quiescent G0 state when re-exposed to higher concentrations of 5FU. These quiescent cells exhibited upregulated expression levels of c-YES/YAP signaling. Moreover, clinical results indicated that the transcript levels of YES1 and YAP are higher in colon cancer patients with liver metastases after 5FU-based neoadjuvant chemotherapy, which was also positively correlated with colon cancer recurrence and shorter patient survival [41]. Further studies showed that 5FU-induced quiescent cancer cells expressed high levels of YAP and decreased levels of cyclin E1 and c-Myc, which were associated with shorter disease-free and overall survival [119]. Taken together, the c-Yes/YAP signaling pathway can be considered as a potential therapeutic target to kill drug-resistant quiescent cancer cells.

(e) Quiescent cancer cells show immune evasion capacity

Metastasis typically occurs years after primary tumor resection, with a small number of disseminated cancer cells surviving as latent entities through unknown mechanisms. The researchers isolated latent carcinoma cells (LCC) from early human lung and breast tumors and found that these LCC cells readily entered a quiescent state in mitogen-low medium (MLM, 2% serum), whereas apoptotic markers (for example, cleaved caspase-3) remained unchanged for months as potential entities in relevant organs. These LCC quiescent cells still retained tumorigenic and metastasis-initiating potential. Further studies revealed that these QCCs expressed protein DKK1, which further leads to the downregulation of NK cell activators and escape from immune surveillance [5]. This finding suggests that selectively reactivating NK cell ligands in quiescent metastatic cells could trigger the immunologic elimination of latent metastasis. With the exception of being able to escape from immune surveillance, the latest study also revealed that QCCs, expressing a high level of quiescent marker p27 in breast tumor cells, were able to resist T cell attack by forming an immunosuppressive niche. These QCCs exhibited high levels of genes related to chemoresistance (Car9, Kdm5a, and Kdm5b), hypoxia (Hif1a), and glycolysis (glucose transporter Slc2a1 or Glut1). However, the expression level of Cd81, Il12a, and Il12b, which represents a key cytokine for T cell responses [120], was lower in the QCC niche, suggesting that QCCs could lead to immunotherapy resistance by orchestrating a local hypoxic immunosuppressive environment to block T cell function, and restoring the function of T cell holds promise to eliminate QCCs and thus counteract immunotherapy resistance [34].

**Figure 2:**
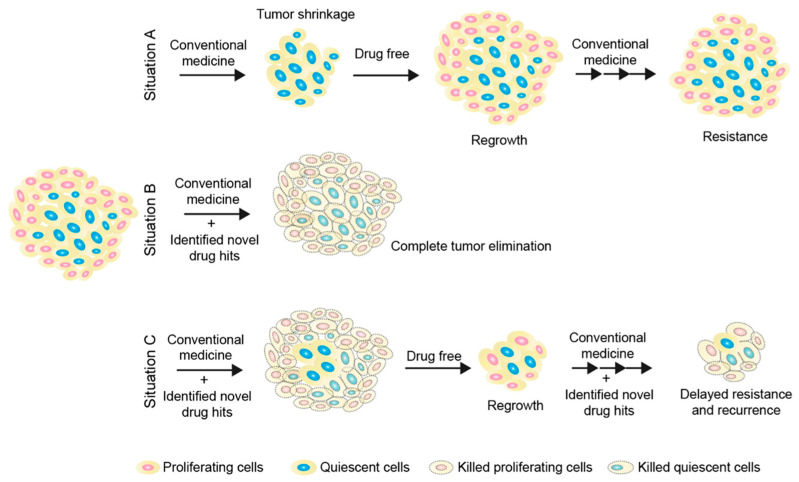
Elimination of tumor cells by targeting both proliferative and quiescent cancer cell populations. Situation (**A**) describes the current clinical obstacle in the treatment of solid tumors due to the existence of quiescent cancer cells, which are insensitive to cell-cycle-active drugs (Figure 1). Situation (**B**) describes an ideal outcome by targeting both proliferative and quiescent cancer cell populations. Briefly, proliferative cancer cells could be targeted by cell-cycle-dependent anticancer reagents and quiescent cancer cells could be removed by small-molecule drugs or immunotherapies developed from strategy 3. Situation (**C**) describes a feasible strategy. In brief, targeting both proliferative and quiescent cancer cell populations has a better chance of removing a larger proportion of tumor cells compared to Situation A. During the drug-free period, residual tumors will regrow, but fewer latent cells could result in smaller regrowth tumors. Re-expanded tumors can be re-treated by targeting both proliferating and quiescent cancer cell populations. This strategy could be able to delay the onset of drug resistance and tumor recurrence, thereby prolonging survival expectations.

A strategy of removing cancer cells while they are in a quiescent state may be effective enough to overcome QCC-induced drug resistance and tumor recurrence (Figure 2). However, achieving this requires the overcoming of several challenges: (i) establishing in vitro and in vivo models that contain quiescent cancer cell populations under different conditions—the different conditions here include, but are not limited to, the microenvironment in the untreated primary tumor, the microenvironment in the primary tumor after certain treatments (mitochondrial inhibitors, autophagy inhibitors, kinase inhibitors, mTOR inhibitors, MEK inhibitors, etc.), or the microenvironment in the metastatic site; (ii) the development of a reliable and sensitive clinical diagnostic tool to detect quiescent cancer cells; and (iii) as QCCs are always located in areas away from solid tumor vessels, the development of highly efficient and specific drug delivery systems is considered another challenge. Despite all the challenges, identifying the unique features of quiescent cells to further eliminate them still holds great potential to overcome chemoresistance and recurrence in solid tumor therapy. Any achievements in this field may ultimately improve cancer treatment outcomes in solid tumors.

## 6. Concluding Remarks and Future Prospects

Quiescent cancer cells are able to avoid most cancer treatments and have been associated with cancer recurrence as they can re-enter the proliferative state when the conditions are beneficial. Accumulated studies aiming to find therapeutic options for QCCs in solid tumors have revealed several cues to overcome resistance and relapse in cancer therapy. Here, we reviewed and discussed the latest research progress of treatments against QCCs and described four therapeutic strategies, including (i) reactivating quiescent cancer cells and remove them via cell-cycle-dependent anticancer reagents; (ii) modulating the quiescence-to-proliferation switch; and (iii) eliminating QCCs by targeting their unique features.

Despite great progress, QCC-targeted therapy remains challenging. First, the lack of reliable QCC models and detection methods limits the research on the mechanisms of QCC activation and survival, as well as the development of QCC-targeting drugs. Second, due to the heterogeneity of QCCs, not all QCCs will respond to the same treatment. Third, current interventions for QCCs may have side effects because of their nonspecific characteristics. These challenges highlight the need to develop more rational QCC models, elucidate the specific molecular mechanisms of QCCs’ activation and survival, and identify more specific targets for QCC therapy. It is believed that co-targeting proliferating and quiescent cancer cells simultaneously could ultimately lead to long-lasting remissions and improved survival for cancer patients (Figure 2, Situation C).

## Figures and Tables

**Table 1:** Overview table of markers for quiescent cancer cells and their function.

Marker Expression Level	Function to Induce Quiescent State
**p27^high^**	Inhibiting CDK 4/6 [15]
**p21^high^**	Inhibiting CDK 4/6 [15]
**ERK1/2^low^ p38^high^**	Upregulation of, for example, NR2F1 and p53 [18]
**NR2F1**	Induction of CDK inhibitors p16 and p27 [19]
**DYRK1A**	Activating DREAM complex [20]
**Mirk/DYRK1B**	Stabilization of p27 and activation of DREAM complex [24,25]
**p130/Rb^high^**	Inhibition of E2F4 transcription factor [23]
**Ki67^low^**	Only associated with cells that are rapidly growing and dividing [16]

## Data Availability

Not applicable.

## Abbreviations

5-FU	5-Fluorouracil
CDKs	cyclin-dependent kinases
CSCs	cancer stem cells
DTCs	disseminated tumor cells
DYRK	dual-specificity tyrosine-regulated kinase
IDH3	isocitric dehydrogenase 3
MCTS	multicellular tumor spheroids
LCC	latent carcinoma cells
OXPHOS	mitochondrial oxidative phosphorylation
QCCs	quiescent cancer cells
Smoi	smoothened inhibitor
